# Gene-activated matrix/bone marrow-derived mesenchymal stem cells constructs regenerate sweat glands-like structure *in vivo*

**DOI:** 10.1038/s41598-017-17967-x

**Published:** 2017-12-15

**Authors:** Pranish Kolakshyapati, Xiuyuan Li, Chunye Chen, Mingxia Zhang, Weiqiang Tan, Lie Ma, Changyou Gao

**Affiliations:** 1 MOE Key Laboratory of Macromolecular Synthesis and Functionalization, Department of Polymer Science and Engineering, Zhejiang University, Hangzhou, 310027 P.R. China; 2 Department of Plastic Surgery, The Fourth Affiliated Hospital, College of Medicine, Zhejiang University, Yiwu, 322000 P.R. China

## Abstract

It is a significant challenge to regenerate full-thickness skin defects with sweat glands. Various skin substitutes have been developed to resolve this issue with minimal success. In this study, to yield a novel construct for *in situ* regeneration of sweat glands, the collagen-chitosan porous scaffold was combined with Lipofectamine 2000/pDNA-EGF complexes to obtain the gene-activated scaffold (GAS), which was then seeded with bone marrow-derived mesenchymal stem cells (BM-MSCs). The porous scaffold functionalized as a reservoir for the incorporated gene complexes which were released in a sustained manner. The seeded BM-MSCs were transfected *in situ* by the released complexes and specially differentiated into sweat gland cells *in vitro* under the induction of the expressed epidermal growth factor (EGF). Application *in vivo* of the GAS/BM-MSCs constructs on the full-thickness skin defects of SD rats confirmed that GAS/BM-MSCs could accelerate the wound healing process and induce the *in situ* regeneration of the full-thickness skin with sweat gland-like structures. Analyzed by immunohistochemical staining, RT-qPCR and Western-blotting, the levels of the major sweat gland markers such as carcino-embryonic antigen (CEA), cytokeratin 8 (CK8) and cytokeratin 14 (CK14) were all up-regulated, indicating that GAS/BM-MSCs can facilitate the regeneration of sweat glands-like structure *in vivo*.

## Introduction

Skin and its appendages such as hair follicles, sebaceous glands, and sweat glands have a high risk of suffering partial or full-thickness damage because of injuries or burns^1,2^. Skin appendages, which are absent in the healed skin, play a significant role in the essential functions of skin such as thermal regulation, sensation, and lubrication^3^. As one of the vital parts of skin appendages, sweat glands perform several functions including secretion of sweat, excretion of wastes, maintenance of body temperature and inhibition of bacterial growth by secretion of lactate^4^. Sweat glands cannot be regenerated in case of full-thickness skin defect, resulting in the loss of perspiration function. To date, there is no effective treatment available for the patients with irreversible loss of functional sweat glands. Hence, it is a huge challenge to regenerate a fully functional skin not only with epidermis and dermis but also skin appendages, especially sweat glands.

Recent solutions include transplantation of sweat gland cells and tissue-engineered skin. Li *et al*. demonstrated that the epithelial cells derived from sweat glands could keep the morphology of sweat gland cells when they are seeded into Matrigel^5^. Likewise, subcutaneous implantation of the eccrine sweat gland cells-seeded Matrigel into nude mice resulted in the formation of tubular-like structures^6^.

However, the sweat gland cells are very few and hard to be harvested. Therefore, several types of stem cells, such as epidermal stem cells, sweat gland stem cells, and mesenchymal stem cells (MSCs) have been applied for the regeneration of sweat glands^4^. Due to the limitation of cell source, the use of sweat gland stem cells meets many realistic difficulties^1,7^. In contrast, MSCs especially bone marrow-derived MSCs (BM-MSCs) are regarded as an important cell source for the regeneration of eccrine sweat glands, as they receive less direct damage from skin injury and possess lower immunogenicity and great expansive potential^1,7,8^. Furthermore, the latest researches have shown that MSCs are capable of being induced to acquire a sweat gland phenotype under a proper microenvironment^9,10^. Therefore, BM-MSCs have significant theoretical and practical value for the regeneration of sweat glands.

Even though the mechanism of sweat gland regeneration by MSCs is still unclear, various cytokines seem to play important regulatory roles in the regeneration and development of sweat glands^4^. One of the key cytokines is epidermal growth factor (EGF), which actively involves in the morphogenesis and homeostasis of skin and sweat glands. Its expression increases gradually in the developing sweat gland bud and the extracellular stroma during embryonic development^11^. Moreover, in adults, it is strongly expressed in the myoepithelial cells of eccrine sweat glands and is positive in the secretory cells and myoepithelial cells of apocrine sweat glands^12^. Hence, a material system is needed to deliver MSCs and EGF signals simultaneously for the regeneration of sweat glands.

It is well-known that the direct application of naked growth factors has limited success due to the short half-life and instability in the physiological environment^13^. To overcome these limits, the application of functional gene encoding the proper growth factor has been evaluated as a feasible strategy. Indeed, the gene-activated scaffold has attracted much attention nowadays^14,15^. The gene-activated scaffold could act as a reservoir for functional genes, facilitating *in situ* gene transfection and expression of the specific biosignals to regulate cell behaviors. They have been successfully applied for the regenerations of bone, cartilage, and skin^15–17^, with improved granulation tissue formation, angiogenesis and reepithelization^18–21^. All these pioneered studies prove that the gene-activated scaffolds may serve as a good platform for inducing the differentiation of MSCs and therefore the regeneration of sweat glands.

A number of materials such as collagen^22–25^, chitosan^26^, fibrin^27^, gelatin^28,29^, and hyaluronic acid^30,31^ have been used to fabricate skin substitutes, in which the collagen-based scaffolds are most widely used^32,33^. In our group, bilayer dermal equivalent (BDE) was constructed by covering the collagen-chitosan scaffold with a silicone membrane, which allowed the reconstruction of dermis in a full-thickness skin defect of Bama miniature pigs^13,26^. Furthermore, BDEs loaded with N,N,N-trimethyl chitosan chloride (TMC)/pDNA-VEGF and TMC/siRNA-TGF-β1 complexes were fabricated to enhance angiogenesis^13^ and inhibit scar formation, respectively^23^. All these results demonstrate that the gene-activated scaffold could serve as an effective platform for skin regeneration.

Encouraged by these previous works, in this study, a kind of gene-activated scaffold (GAS) is fabricated by loading the Lipofectamine 2000/plasmid DNA-encoding EGF (pDNA-EGF) complexes into the collagen-chitosan porous scaffold. Then, BM-MSCs are cultured into GAS to build a construct. The physiological and biological properties of the GAS/BM-MSCs constructs (GAS/BM-MSCs) are further examined. The differentiation potential of BM-MSCs into sweat gland cells is evaluated at both gene and protein levels *in vitro*. Furthermore, the *in vivo* tests are carried out by transplanting the GAS/BM-MSCs constructs onto the hind paws of Sprague-Dawley (SD) rats to assess their ability in regenerating skin with sweat gland-like structures.

## Results

### Morphology of GAS/BM-MSCs constructs

**Figure 1:**
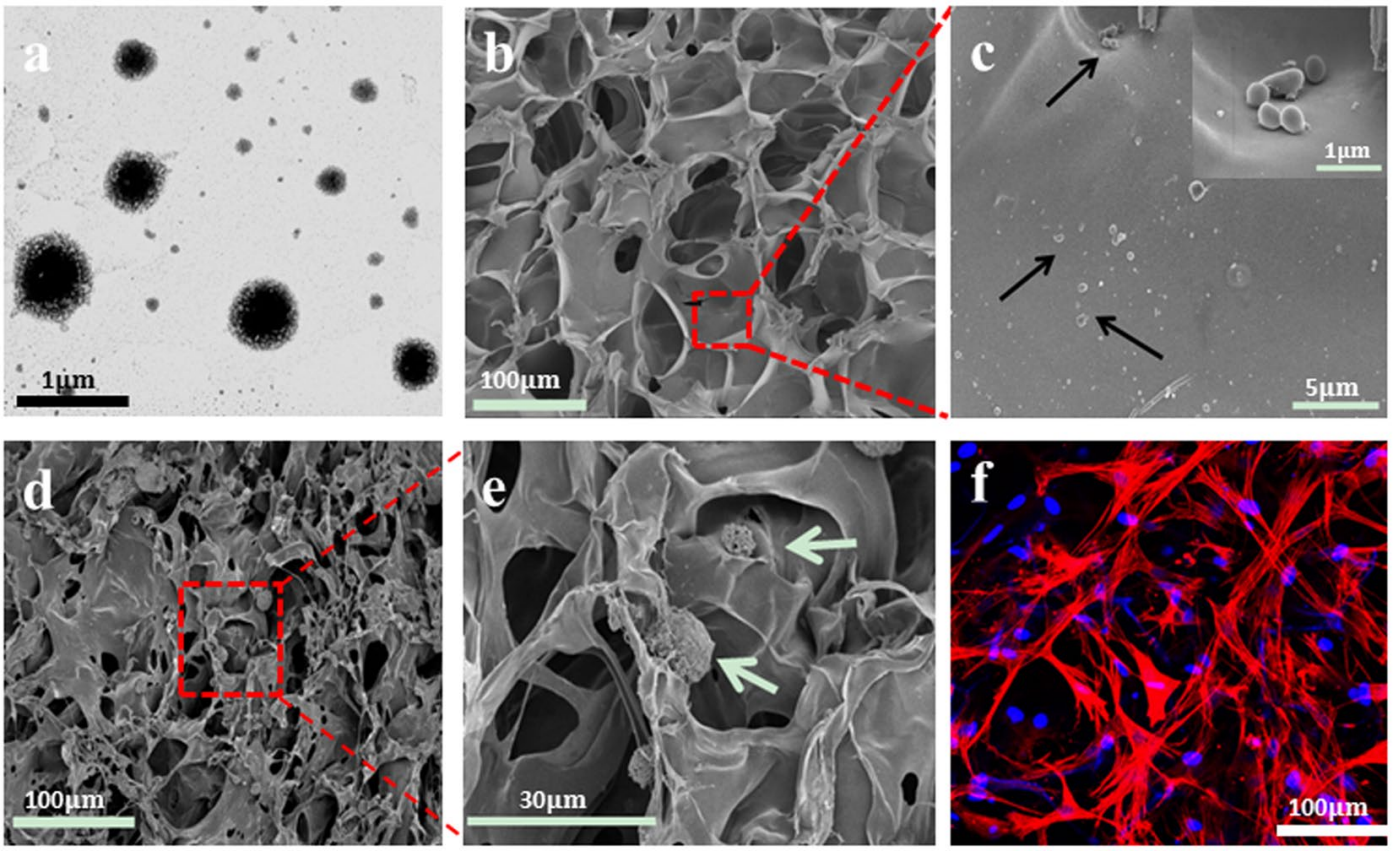
(**a**) TEM image of Lipofectamine 2000/pDNA-EGF complexes. (**b**,**c**) SEM images of Lipofectamine 2000/pDNA-EGF complexes-loaded collagen-chitosan scaffold (GAS) with different magnifications. The inset image in (**c**) shows the enlarged view of the highlighted area. Black arrows indicate the Lipofectamine 2000/pDNA-EGF complexes. (**d**,**e**) SEM images of GAS/BM-MSCs construct with different magnifications. Green arrows represent the BM-MSCs cultured on GAS. (**f**) Confocal laser scanning microscopy image of GAS/BM-MSCs construct after being cultured for 7 days, in which nucleus was stained with DAPI (Blue), and cytoskeleton was stained with rhodamine phalloidine (Red).

The Lipofectamine 2000/pDNA-EGF complexes had a regular spherical shape with a diameter of 300–400 nm observed under TEM (Fig. 1a). The GAS demonstrated an open pore microstructure and a high degree of interconnectivity with a pore size of 100–150 µm (Fig. 1b). The gene complexes were attached to the scaffold walls from the image of higher magnification (Fig. 1c), suggesting the successful loading. The seeded BM-MSCs adhered well on the scaffold surface or penetrated into the scaffold along the pores (Fig. 1d,e). The morphology of BM-MSCs with the co-staining of cell nucleus and cytoplasm was observed clearly under CLSM (Fig. 1f), which showed the well spreading morphology.

### DNA release and *in vitro* EGF expression

**Figure 2:**
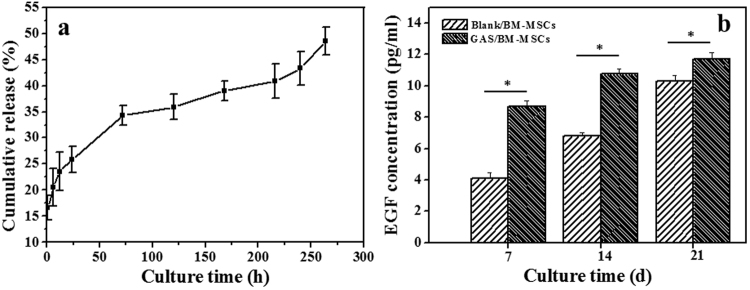
(**a**) Cumulative release of DNA from GAS as a function of time in PBS at 37 °C. (**b**) ELISA analysis of EGF concentration in Blank/BM-MSCs and GAS/BM-MSCs constructs after being cultured for 7, 14 and 21 days *in vitro* (n = 3). * denotes statistically significant difference at p < 0.05.

The *in vitro* release behavior of DNA from the collagen-chitosan scaffold was investigated (Fig. 2a). The DNA was released up to 25.9 ± 2.5% in the first 24 h, and then released in a relatively steady pattern. 35.9 ± 2.4% and 48.5 ± 2.7% DNA were released at 120 h and 260 h, respectively. Hence, the GAS can enable the sustained release of the incorporated DNA efficiently for a long period. Furthermore, from the results shown in Supplementary Fig. S10, it is proved that the released DNA complexes at day 9 still have the ability to transfect BM-MSCs, although the transfection efficiencies decreased with the releasing time.

Figure 2b reveals that the expression levels of EGF increased with the culture time prolongation in both GAS/BM-MSCs and Blank/BM-MSCs constructs *in vitro*. However, the levels of GAS/BM-MSCs were significantly (p < 0.05) higher than those of the Blank/BM-MSCs at all the time points, demonstrating that the incorporation of pDNA-EGF could enhance the EGF expression.

As presented in Supplementary Fig. S2, the absorbance values at 570 nm increased with culture time, which is mostly attributed to the increased number of viable cells. The GAS/BM-MSCs had a similar level of viability as that of the Blank/BM-MSC constructs at day 1. The viability of both groups was comparable at day 3, while the GAS/BM-MSCs group had slightly higher value at day 7. These results reveal that the GAS was suitable for supporting the proliferation of BM-MSCs and showed no obvious cytotoxicity.

### *In vitro* differentiation of BM-MSCs into sweat gland cells

**Figure 3:**
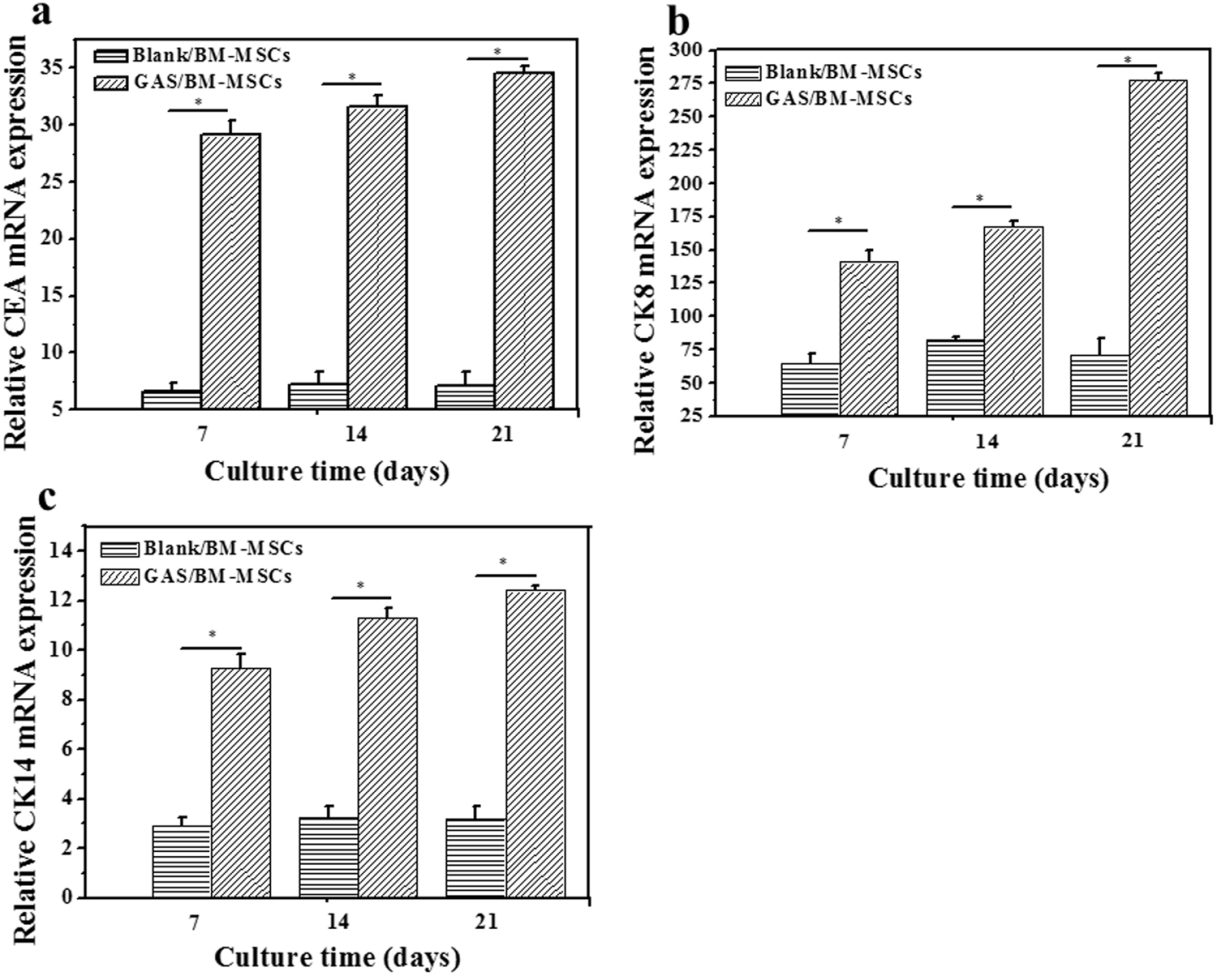
RT-qPCR analyses of mRNA expression for (**a**) CEA, (**b**) CK8 and (**c**) CK14 by Blank/BM-MSCs and GAS/BM-MSCs constructs after being cultured for 7, 14 and 21 days *in vitro*, respectively. * denotes statistically significant difference at p < 0.05.

RT-qPCR was used to quantify the *in vitro* mRNA expression of CEA, CK8, and CK14, which are the key markers of sweat gland cells (Fig. 3). The mRNA expression of GAS/BM-MSCs for all the three markers was significantly (p < 0.05) higher than those of the Blank/BM-MSCs at all the time points (Fig. 3a–c). The expression levels kept almost constant for the Blank/BM-MSCs at each time point, whereas those of the three markers in the GAS/BM-MSCs increased along with time prolongation. These results suggest that the BM-MSCs cultured in GAS were differentiated towards sweat gland cells phenotype and thus showed abundant expression of the sweat gland markers.

**Figure 4:**
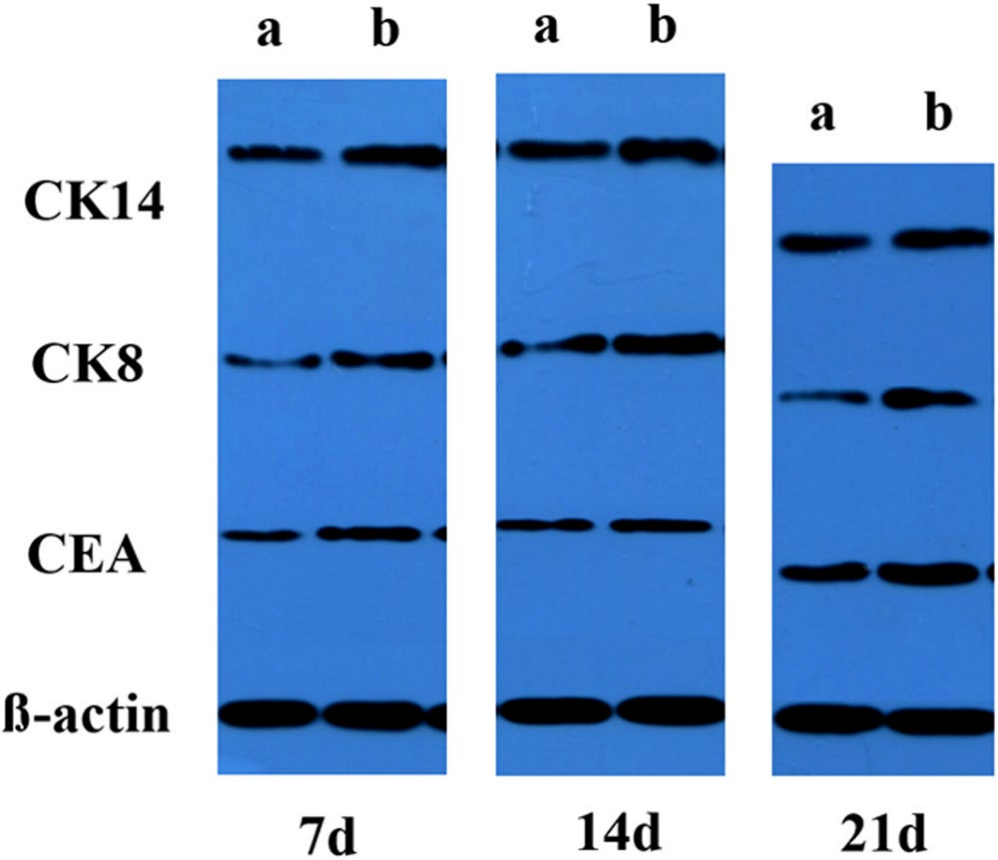
Western blotting analysis of CEA, CK8 and CK14 expressed by BM-MSCs cultured in (**a**) Blank/BM-MSCs and (**b**) GAS/BM-MSCs constructs for 7, 14 and 21 days *in vitro*, respectively. For these three western blotting experiments (7d, 14d and 21d) were performed at different time, the blot images were difficult to be aligned horizontally.

Western blotting analysis was carried out to detect the contents of CEA, CK8 and CK14 at the protein level (Fig. 4). The CEA bands of GAS/BM-MSCs were apparently more intense than those of the Blank/BM-MSC at all the time intervals, and became most intense at day 21. Similarly, the CK8 bands of GAS/BM-MSCs were always more intense as compared to those of the Blank/BM-MSCs at all the time points. The CK14 bands of GAS/BM-MSCs did not show that much difference as the other two markers, but were still intense at each time points, as revealed by the densitometry analysis (Supplementary Fig. S3).

A larger number of CK8 and CK14-positive cells in the GAS/BM-MSCs group were found in the cultured constructs *in vitro* as examined by immunohistochemical staining (Supplementary Fig. S4). In some regions, they were densely aggregated, mimicking the structure of sweat gland-like cells especially at day 21 (Supplementary Fig. S4b,d,f,h,i,j). Compared to the GAS/BM-MSCs group, relatively weaker staining and fewer of aggregated structures could be observed in the Blank/BM-MSCs group (Supplementary Fig. S4a,c,e,g,i,k).

### Macroscopic appearance of wounds

**Figure 5:**
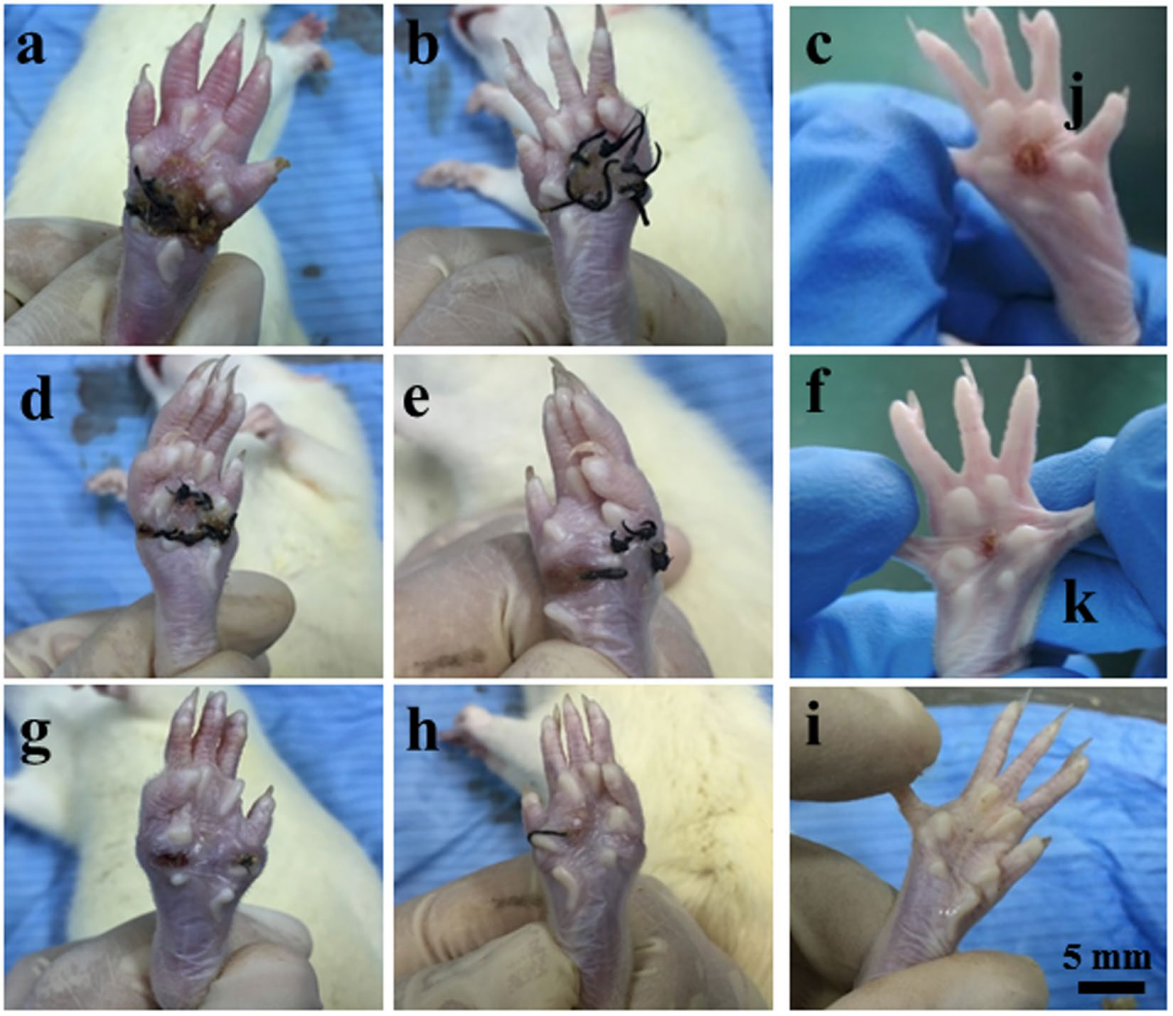
Macroscopic appearance of the wounds treated by GAS (**a**,**d**,**g**), Blank/BM-MSCs (**b**,**e**,**h**) and GAS/BM-MSCs constructs (**c**,**f**,**i**) for 2 w (a,b,c), 4 w (d,e,f), and 8 w (g,h,i) post-surgery, respectively.

Figure 5 shows the macroscopic appearance of the wounds treated with different groups of samples for 2, 4 and 8 w. At 2 weeks post-surgery, a better closure was observed on the GAS/BM-MSCs-treated wounds compared with the wounds treated by GAS and Blank/BM-MSCs (Fig. 5a–c). The wound was closed much more quickly with better and smooth surface and less inflammation in the GAS/BM-MSCs group 4 w post-implantation (Fig. 5f). The wounds in the control groups healed slowly, and the obvious inflammation on the wound surface was visible (Fig. 5d,e). At week 8, the wound was almost repaired, and no obvious contraction was observed in the group of GAS/BM-MSCs (Fig. 5i). However, there were different degrees of contraction and evident inflammation in the GAS and Blank/BM-MSCs treated wounds (Fig. 5g,h). Thus, GAS/BM-MSCs not only accelerated wound closure but also achieved wound healing without obvious inflammation, and the regenerated skin showed a remarkable similarity to the natural skin. In addition, pictures of the healthy paw and the freshly wounded paw were provided in Supplementary Fig. S5.

### Histology

**Figure 6:**
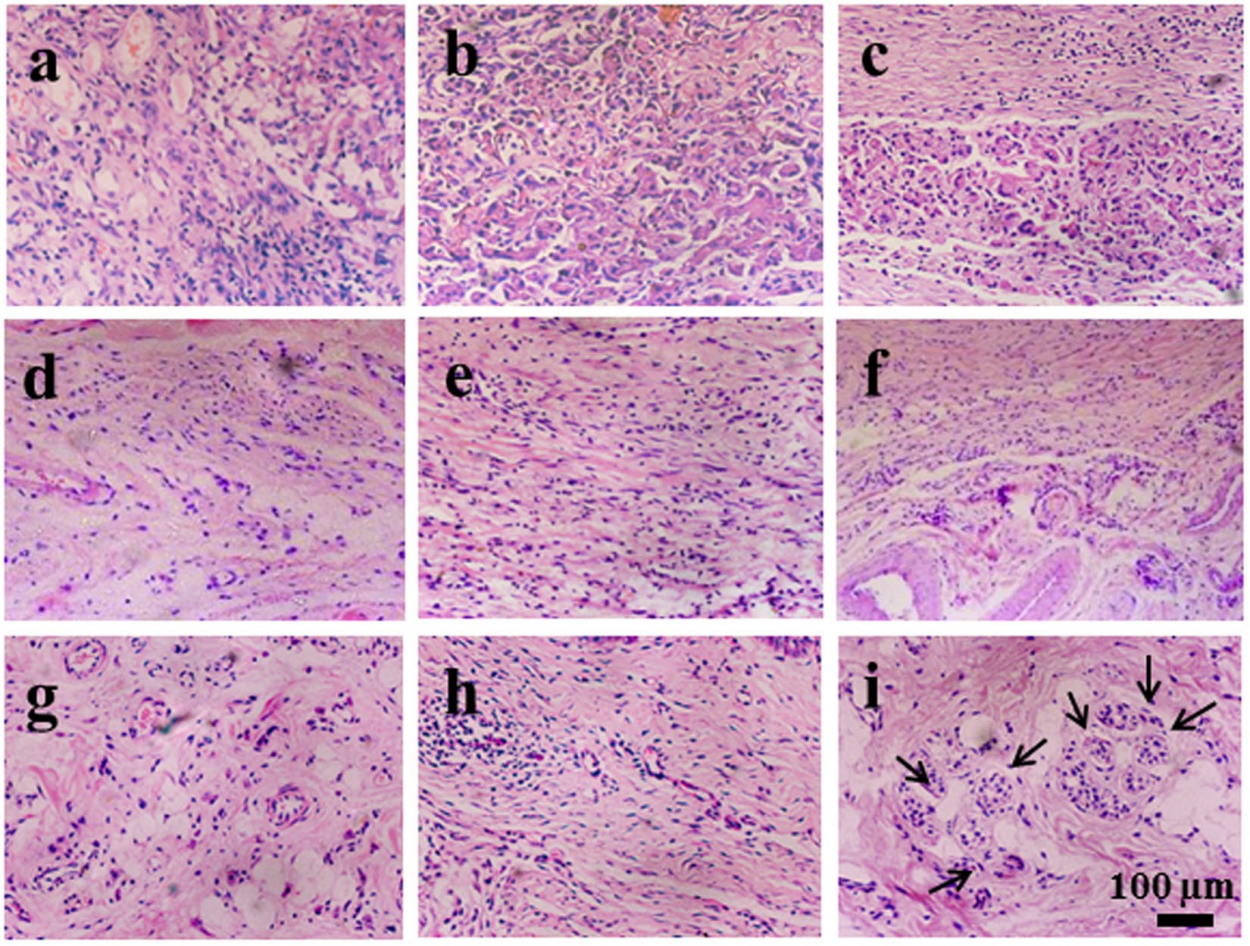
H&E staining of sections of wounds treated by GAS (**a**,**d**,**g**), Blank/BM-MSCs (**b**,**e**,**h**) and GAS/BM-MSCs constructs (**c**,**f**,**i**) for 2 w (a,b,c), 4 w (d,e,f) and 8 w (g,h,i) post-surgery, respectively. Arrows indicate sweat gland-like structures.

The biopsies of the wounds treated by different groups for 2 W, 4 W, and 8 W were collected for histological analysis (Figs 6 and S6). At week 2 (Fig. 6a–c), a number of inflammatory cells and emergence of granulation tissue could be observed in all of the groups (Supplementary Fig. S6a–c and Fig. S6). Moreover, the discontinuous epidermis was observed clearly, indicating the unclosed wound state (Supplementary Fig. S7). At week 4, the inflammation response began to subside, and skin repair progression could be observed with the gradual evolvement of dermal and epidermal ridges (Supplementary Fig. S6d–f). Many depositions of extra cellular matrix (ECM) could be seen in GAS/BM-MSCs compared to the other two control groups, although no neonatal sweat gland-like structures could be observed in any group (Fig. 6d–f). At week 8, the HE staining results of GAS/BM-MSCs revealed better dermis regeneration along with tubular coiled structures, which were not visible in the other two groups (Supplementary Fig. S6g–i and Fig. 6g–i). These results suggest that GAS/BM-MSCs could effectively promote skin repair with obvious sweat gland-like structures, similar to those in the natural skin (Supplementary Fig. S8).

### Immunohistochemical analysis

Immunohistochemical analysis was performed to identify the expression of the sweat gland specific markers. The commonly used markers of sweat glands include CK8, CK14, and CEA. Although CEA is known to be expressed in sweat glands, the positive staining was not achieved in any group. CK14 is expressed more in ductal epithelial cells in sweat glands and weakly expressed in basal myoepithelial cells, whereas CK8 is expressed in the secretory unit of sweat glands.

**Figure 7:**
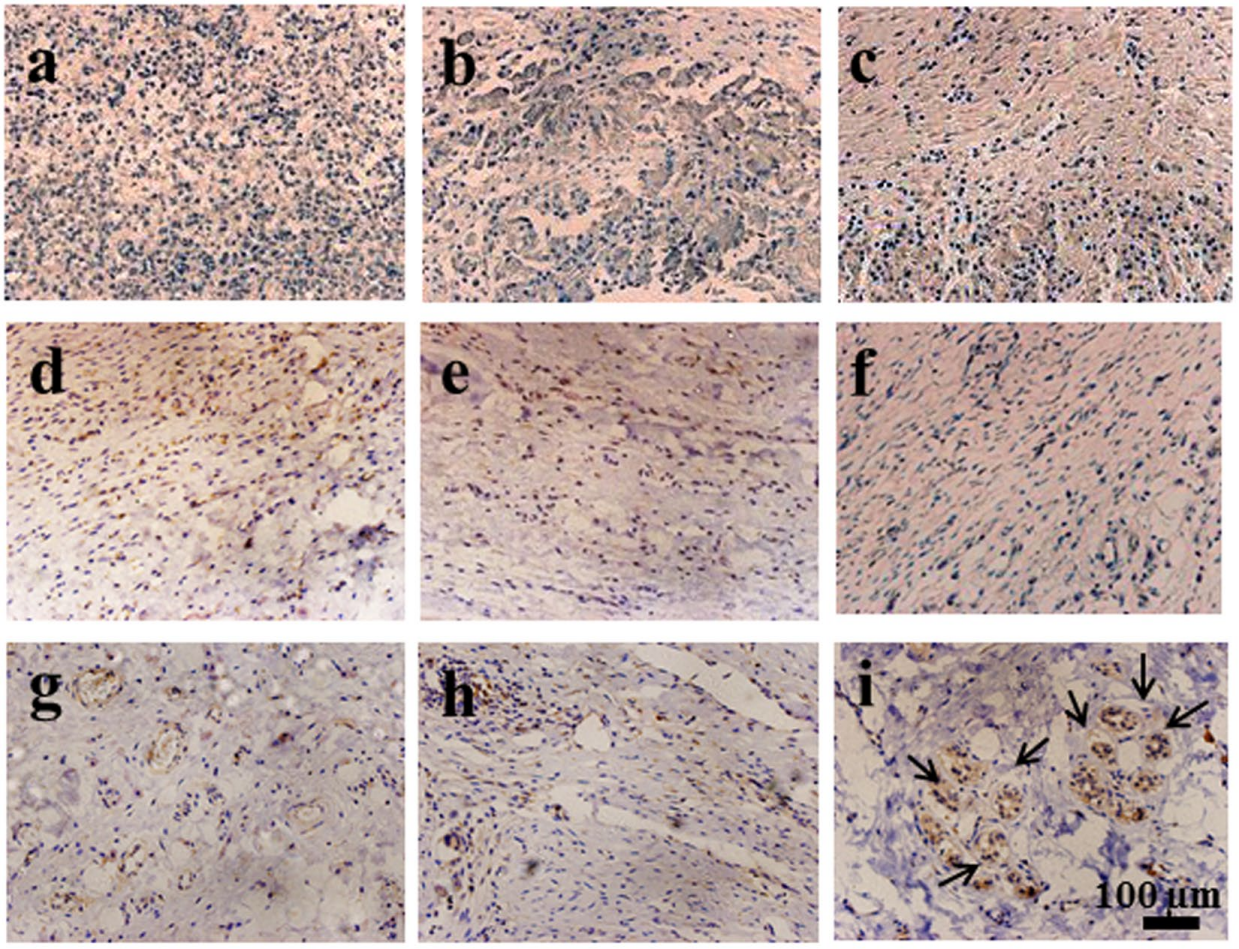
Immunohistochemical staining of CK8 on sections of wounds treated by GAS (**a**,**d**,**g**), Blank/BM-MSCs (**b**,**e**,**h**) and GAS/BM-MSCs constructs (**c**,**f**,**i**) for 2 w (a,b,c), 4 w (d,e,f), and 8 w (g,h,i) post-surgery, respectively. Arrows indicate sweat gland-like structures.

**Figure 8:**
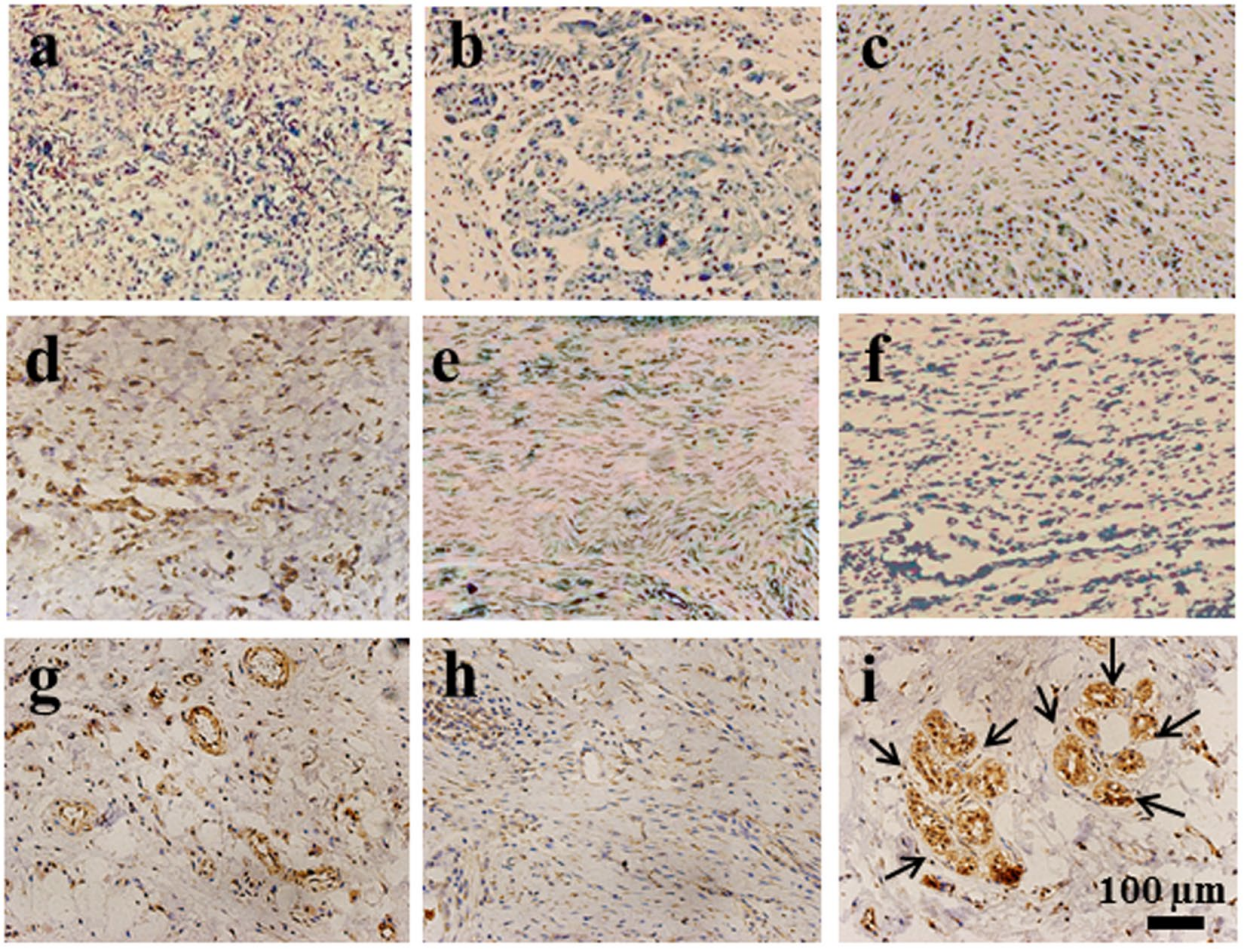
Immunohistochemical staining of CK14 on sections of wounds treated by GAS (**a**,**d**,**g**), Blank/BM-MSCs (**b**,**e**,**h**) and GAS/BM-MSCs constructs (**c**,**f**,**i**) for 2 w (a,b,c), 4 w (d,e,f), and 8 w (g,h,i) post-surgery, respectively. Arrows indicate sweat gland-like structures.

As shown in Fig. 7, the positive staining of CK8 was observed for all the tissue samples regardless of the implantation time, but its level depended greatly on the group and the implantation time. At week 2 and week 4, there was no significance difference in the CK8 staining between the GAS/BM-MSCs and the other two groups (Fig. 7a–f). After transplantation for 8 w, the aggregated sweat gland-like structures could be observed in the sections of GAS/BM-MSCs, which expressed stronger positive staining than the other two groups (Fig. 7g–i). Likewise, CK14 was also stained positively for all the groups at the given time points, and the staining was stronger at week 4 (Fig. 8a–c) than those at week 2 (Fig. 8d–f) for all groups. The GAS/BM-MSCs and Blank/BM-MSCs showed stronger staining than the GAS at week 4 (Fig. 8d–f). Furthermore, 8 w after implantation, the sweat glands-like structures were strongly stained and could be observed clearly in the GAS/BM-MSCs group (Fig. 8i).

### RT-qPCR analysis

**Figure 9:**
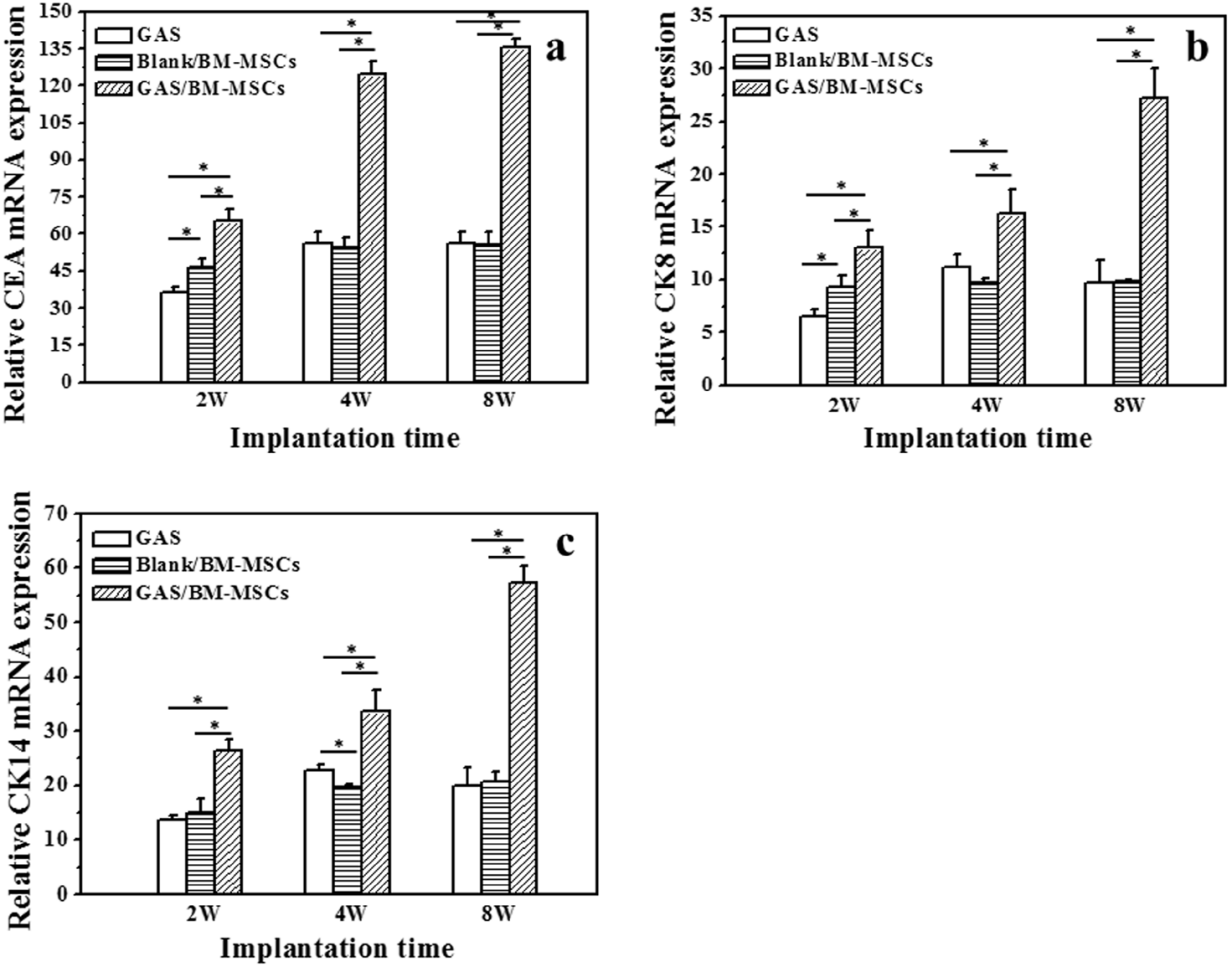
Real-time quantitative PCR (RT-qPCR) analyses of mRNA for (**a**) CEA, (**b**) CK8, and (**c**) CK14 in tissue sections of rat wounds after treated by GAS, Blank/BM-MSCs, and GAS/BM-MSCs constructs for 2 W, 4 W and 8 W post-surgery, respectively. * denotes statistically significant difference at p < 0.05.

The *in vivo* expression of the key markers, i.e. CEA, CK8, and CK14, were further analyzed using RT-qPCR. The mRNA expressions of CEA, CK8 and CK14 in GAS/BM-MSCs showed the similar patterns, which increased with the time prolongation (Fig. 9). A marked increase at the mRNA level was found at week 4 for CEA (Fig. 9a), whereas the significant increases (p < 0.01) were noticed at week 8 for CK8 and CK14 (Fig. 9b–c). Comparatively, the expression levels of the other two groups were relatively lower at all the time points, without noticeable change too. The results imply that the BM-MSCs cultured in GAS/BM-MSCs were induced to differentiate into sweat gland cells and further to promote the regeneration process of sweat gland-like structures *in vivo*.

### Western-blotting analysis

**Figure 10:**
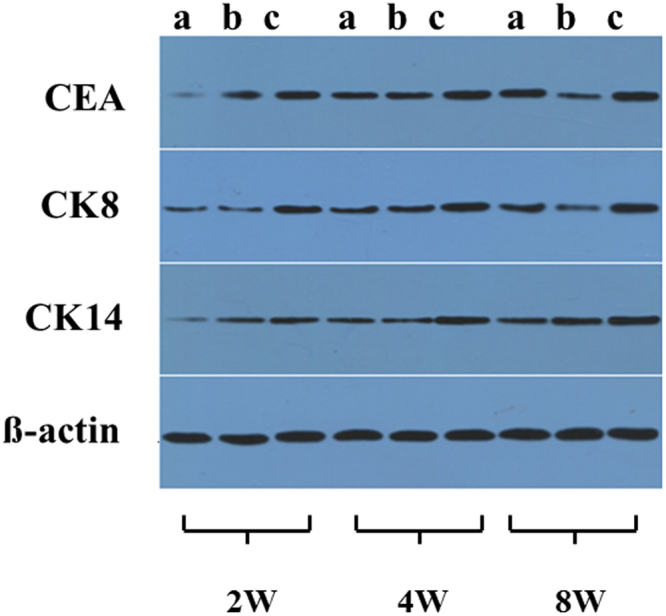
Western blotting analysis of CEA, CK8, CK14 from tissue sections of rat wounds after treated by GAS (**a**), Blank/BM-MSCs (**b**), and GAS/BM-MSCs constructs (**c**) for 2 W, 4 W, and 8 W post-surgery, respectively.

The sweat gland markers were further analyzed at protein level by WB (Figs 10 and S9). The GAS/BM-MSCs had the highest protein expression levels at week 2 for all the markers. Noticeably, GAS had the faintest bands, and thus the lowest expressions of all the three markers at this time point. Likewise, at week 4 and week 8, GAS/BM-MSCs had the most intense bands among three groups. This finding is consistent with the above results, highlighting the effective role of GAS in the differentiation of BM-MSCs into sweat gland cells and their efficient contribution for the development of sweat glands-like structures in the healed skin.

## Discussion

Skin is an important organ of human body serving a range of regulatory and mechanical functions. Although the existing skin substitutes, such as autografts, allografts and tissue engineering skin *et al*. can heal the damaged skin to a certain extent, the vital skin appendages like hair follicles, sweat glands, and sebaceous glands are normally missing. Among these appendages, sweat gland, being an indispensable part of skin, is important for temperature regulation, excretion of wastes and maintenance of skin homeostasis. Thus, the inability of the current methods to regenerate a fully functional skin with sweat glands has been a significant issue. Although numerous attempts have been made to advance the feasibility and clinical applicability of sweat gland regeneration, effective and optimal methods have not yet been well developed^36–38^.

The major focus of the current study is to disclose the influence of local *in vivo* microenvironment on the skin regeneration of a GAS/BM-MSCs construct, namely, the regeneration effects and the types of regenerated tissues depend not only on the construct itself, but also on the local niche. In this way, we loaded only one type of genes (pDNA-EGF) into the construct, and did not conduct *in vitro* induced differentiation of GAS/BM-MSCs construct toward the sweat glands. This is a very new concept to study the adaptive regeneration behaviors of implanted construct, with many possibilities and uncertainties of the regeneration outcomes. In this regard, the *in vitro* studies are not able to give definite answer if the current construct is able to regenerate the sweat-gland like structures *in vivo*, although the basic results reveal the possibility of local gene transfection and, as a result, secretion of related proteins.

With the emergence of stem cell biology and regenerative medicine, stem cells have been widely used for the regeneration of many kinds of tissues^10,39^. The cell therapy with BM-MSCs holds enormous promise for the treatment of a large number of diseases. The role of BM-MSCs during wound healing and dermal regeneration has also been extensively studied^10^. BM-MSCs have been found to repair tissue injury by three different mechanisms: the creation of a milieu that enhances regeneration of endogenous cells, transdifferentiation, and cell fusion^40,41^. Due to the immense potential for differentiation and easy expansion *ex vivo* than those stem cells from many other tissues, the BM-MSCs were used in this study.

However, the induction process is rather complicated and easily influenced by many factors. Until now, neither a proper induction medium nor the clear mechanism of sweat gland regeneration from BM-MSCs has been identified. Proper microenvironment for BM-MSCs differentiation is very essential. Among them, the incorporation of desired growth factors during the induction process is considered critical for the differentiation of BM-MSCs into sweat gland cells. To this day, researchers have attempted to induce BM-MSC differentiation into sweat gland cells *in vitro* by adding different kinds of induction factors such as EGF^42^ and ectodysplasin A (EDA)^43^. In particular, EGF is an important growth factor that stimulates re-epithelialization and augments wound healing. It is involved in regulating keratinocyte proliferation at the wound edges and acts on epidermis as mitogens to drive wound closure. As one of the key mitogenic factors for sweat gland development^44,45^, we hypothesized that EGF could not only accelerate wound healing but also induce the differentiation of BM-MSCs into sweat gland cells, assuming that the *in vivo* environment could ultimately provide cues for further regeneration of sweat glands.

However, the delivery of growth factors is still an issue due to their sensitivity and instability, and short half-lives that are only in the order of minutes in serum. An alternative strategy to use plasmid DNA encoding the required growth factor has been proposed^46^. To overcome the drawbacks of low cell transfection efficiency of the naked DNA, gene vectors like synthetic lipids and cationic polymers are widely used^47^. In this study, a commercial non-viral carrier – Lipofectamine 2000 was adopted to realize a high transfection of plasmid DNA and therefore the high expression of EGF. The successful incorporation of the Lipofectamine 2000/pDNA-EGF nanoparticles onto the scaffold was stabilized by the electric interactions and surface adsorption. Furthermore, the proper delivery of the genes is also of paramount importance. The gene-activated scaffold has been developed as a platform for gene delivery and as a reservoir for localized and sustained expression of the growth factors^48^. Moreover, higher transfection efficiency and prolonged expression can be achieved through the plasmid DNA delivered from gene-activated scaffold compared to direct injection^49^. Thus we opted to use GAS loaded with BM-MSC to form the constructs, because the sustained expression of growth factors can better mimic the microenvironment for BM-MSC differentiation. Two prominent sweat gland markers CK8 and CK14 were over expressed, demonstrating the efficacy of the strategy. Cytokeratin is the main structural protein in epithelial cells, and certain cytokeratins act as markers for identifying sweat glands. CK14 is the immunophenotypic marker of differentiation expressed in the inner layer of the sweat duct and usually is expressed in the luminal and peripheral cells of excretory ducts. Likewise, CK8 is expressed in the secretory coils and is a phenotype of organized glandular epithelium^50^. Once the cells differentiate into sweat gland cells, they should gain luminal epithelial markers (CK8)^51^, which were illustrated in our experiment. No positive staining of CEA was observed *in vitro* for any sample, which may be attributed to the inefficiency of CEA antibody or the inaccurate working ratio in the staining procedures.

The microenvironment *in vivo* differs vastly from the *in vitro*. Therefore, the regeneration properties after *in vivo* transplantation serve as a better footprint for the translational clinical application. As sweat glands are restricted to foot paw pads in rats, we established an *in vivo* model system by creating wounds in both hind paws of SD rats for evaluating the GAS’s contribution in skin repair and sweat gland regeneration. The GAS/BM-MSCs had a remarkable effect in accelerating the closure of wound as compared to the controls. It is hypothesized that inflammation may restrict regeneration by promoting fibrosis and scar formation. Though still unclear, it is possible that preventing scar formation is a prerequisite for skin appendage regeneration^52,53^. Thus, minimal inflammation during wound healing may have attenuated scar formation in our study ultimately, leading to enhanced skin reconstruction and sweat gland regeneration. Although it is uncertain whether the regeneration of sweat gland-like structures after transplantation of the GAS/BM-MSC is solely due to the differentiation of the seeded BM-MSCs or due to induction of endogenous stem cells in the implanted region, tubular coiled aggregated structures resembling the sweat glands were obvious only in the experimental group and were stained strongly, signifying that the experimental group may have successfully provided the necessary chemical and physical milieu to mimic the environmental niche for BM-MSC differentiation into sweat gland cells.

The outcome of this study is promising and can provide a feasible strategy for achieving enhanced skin regeneration and sweat gland restoration. Although the direct or indirect contribution of the transplanted BM-MSCs in sweat gland formation is unclear, the results of the GAS/BM-MSCs group were significantly better than the GAS only and Blank/BM-MSCs group, suggesting that the wound healing and the regeneration of sweat gland-like structures might be the outcome of synergistic effect of the loaded pDNA-EGF and the seeded BM-MSCs.

In summary, our current results are sufficient to demonstrate the regeneration of sweat gland-like structures in the regenerated tissues, which is a very important step towards the adaptive regeneration of tissues with multi-types of structures by using the undifferentiated construct, confirming the success and the important value of the current concept. Additional works need to be carried out to extrapolate these findings and ascertain the mechanisms, and to explore the functionalities of the regenerated tissues, for example, the sweat glands.

## Conclusion

This study represents a rational scaffold and gene delivery design to enhance skin wound healing and promote specific differentiation of BM-MSCs into sweat gland cells to realize the regeneration of sweat gland-like structures. The *in vitro* and *in vivo* results are promising, and may have clinical and translational implications in regenerating sweat glands. In conclusion, the gene-activated collagen-chitosan scaffold acts not only as an efficient scaffold for BM-MSC seeding but also as an effective gene delivery vehicle. Synergistic effect of the combination of functional plasmid and appropriate stem cells could prove to be a useful technique for the regeneration of skin with appendages such as sweat glands as shown in this study.

## Methods

### Materials

Lipofectamine 2000 was purchased from Invitrogen Corporation (Carlsbad, CA). DNA (fish sperm) was bought from Sigma (Sigma, MO, USA). Plasmid DNA encoding rat epidermal growth factor (pDNA-EGF) was purchased from Haogene Biotech Co. Ltd (Hangzhou, China). The plasmids were amplified in E.coli and purified by Axygen Maxiprep Extraction kit (Axygen Biosciences, CA, USA) and stored at −20 °C before use. Chitosan (molecular weight 250 kDa, deacetylation degree 85%) was obtained from Haidebei Co., Ltd (Qingdao, China). Silicone membrane was a medical grade product from Shanghai Xincheng Co. Ltd (Shanghai, China). Collagen type I was isolated from fresh bovine tendon by the method of trypsin digestion and acetic acid dissolution in our laboratory^24^. Other chemicals were of analytical grade and used as received. The water used in the experiments was purified by a Milli-Q water system (Millipore, USA).

### Preparation of Lipofectamine 2000/pDNA-EGF complex

A high concentration of pDNA-EGF solution was diluted with Opti-Minimum Essential Media (MEM) into a concentration of 500 µg/mL. Then, the resulting solution was mixed with Lipofectamine 2000 at a volume ratio of 1:1. The mixture was vortexed gently for 3 min, and further incubated for 20 min at 37 °C to form the Lipofectamine 2000/pDNA-EGF complexes. The morphology of the complexes was observed by transmission electron microscopy (TEM, JEOL JEM-200, Japan).

### Fabrication of GAS

GAS was fabricated following the method described previously^13^. Briefly, collagen and chitosan at a mass ratio of 9:1 were dissolved in 0.5 M acetic acid solution to make a mixture with a total concentration of 0.5% (w/v). Then the collagen-chitosan solution was cross-linked by 2.5% (w/v) glutaraldehyde at a final concentration of 0.25% (w/v), and then kept at 37 °C for 4 h. The resulting mixture was injected into a mold, frozen at −20 °C overnight, and then lyophilized for 24 h. The obtained collagen-chitosan scaffolds were sterilized in 75% (v/v) ethanol for 30 min before washed with sterilized phosphate buffered saline (PBS) several times to displace ethanol. Finally, the suspension of Lipofectamine 2000/pDNA-EGF complexes was injected into the scaffold evenly with a multi-point injection method to obtain the GAS after being incubated at 37 °C for 4 h.

### Preparation of GAS/BM-MSCs constructs

Bone marrow-derived mesenchymal stem cells (BM-MSCs) were isolated from bone marrow of Sprague-Dawley (SD) rats (120 g, 6–8 weeks old) according to the methods reported previously^34,35^. The animals for BM-MSCs isolation were approved by the committee on animal experimentation of Zhejiang University and used in accordance with the guidelines for animal experimentation. Briefly, after cervical dislocation of the rats, the hind limbs were dissected. The muscles and connective tissues were carefully removed before the bone marrow was harvested by flushing femurs and tibias with complete medium (α-MEM supplemented with 10% fetal bovine serum (FBS, Gibco, Life Technologies, New York, USA), 100 µg/mL penicillin and 100 U/mL streptomycin) into the marrow cavity. Then the harvested cells were cultured in a 9 cm cell culture dish (Corning, USA) and incubated at 37 °C under 5% CO_2_. The non-adherent cells were removed by 2–3 washes with PBS (pH 7.4) 3 d later, and the adherent cells were further cultured by fresh medium. The harvested cells at 80–90% confluency at passage 2–3 were used in this study, and characterized by flow cytometry (FACSCalibur, BD Bioscience) for differentiation performance *in vitro* (Supplementary Fig. S1).

BM-MSCs were seeded into the blank collagen-chitosan scaffolds (Blank) and GAS to obtain Blank/BM-MSCs and GAS/BM-MSCs constructs (with a diameter of 5 mm and thickness of 2 mm), respectively. For SEM analysis, each scaffold was seeded with 100 μL cell suspension with a concentration of 1 × 10^6^ cells/mL. For other experiments, about 4 × 10^5^ cells were seeded into each scaffold. After 4 h incubation, 500 μl of culture medium was added to immerse the scaffold completely. The constructs were cultured at 37 °C under 5% CO_2_, and the culture medium was changed every 3 d.

### Characterization of GAS/BM-MSCs constructs

For SEM analysis, the GAS/BM-MSCs constructs were washed with PBS and then fixed in 4% (w/v) paraformaldehyde/0.01 M PBS solution (pH 7.4) at 4 °C for 2 h. After the constructs were washed with PBS, they were dehydrated through water-ethanol gradient solutions and ethanol-isobutanol solution, and then were dried by lyophilization. The specimens were coated with gold and examined by SEM (Hitachi, S-3000N, Japan) with an accelerating voltage of 25 kV.

For confocal laser scanning microscopy (CLSM, SP5, Leica, Germany) observation, the GAS/BM-MSCs constructs were rinsed 3 times with PBS and fixed in 4% paraformaldehyde/0.01 M PBS solution for 30 min at 37 °C. The constructs were rinsed 3 times with PBS and further treated in 0.5% (v/v) Triton X-100/PBS at 4 °C for 10 min. After washed with PBS 3 times, they were incubated in 1% BSA/PBS at 37 °C for 30 min. The constructs were rinsed with PBS again and stained with DAPI (4,6-diamidino-2-phenylindole) and rhodamine phalloidine to visualize the nucleus and cytoskeleton, respectively.

### DNA release from GAS

The DNA release behavior from the GAS was examined by conducting an *in vitro* release assay by immersing the GAS into 3 mL of sterile PBS at 37 °C. At the scheduled time intervals, 200 µL of the supernatant was collected for analysis, and the same volume of fresh PBS was replenished. The quantity of the released DNA was tested by a fluorometer (LS55, Perkin-Elmer UK) with Quant-iT PicoGreen assay according to the manufacturer’s instruction (PicoGreen dsDNA Quantitation Kit, Invitrogen, USA). Three parallel samples were carried out at each time point.

### *In vitro* EGF expression

EGF expression of BM-MSCs in the constructs *in vitro* was examined by enzyme-linked immunoabsorbent assay (ELISA) as described previously^16^. At the scheduled time intervals, the constructs were washed three times with PBS (pH 7.4) and then homogenized in the lysis buffer (0.1 M Tris-HCl, 2 mM EDTA, 0.1% Triton X-100). About 2 mL lysate was then centrifuged at 12,000 rpm at 4 °C for 5 min. The supernatant was collected, and the amount of EGF was determined by rat EGF ELISA Kit (70-E-EK393, Multisciences, China) according to the manual. Three parallel samples were carried out at each time point.

### Cell viability assay

The viability of the seeded BM-MSCs was analyzed by MTT assay. Briefly, 4 × 10^5^ BM-MSCs were seeded into Blank and GAS, respectively and incubated for 1, 3 and 7 days. At each interval, the culture medium was aspirated, and then the constructs were washed twice with PBS. 600 µL of pre-warmed culture medium supplemented with 60 µL MTT solution (2.5 mg/mL MTT/PBS) was added to each sample and further incubated for 4 h at 37 °C. Then the medium was removed, and the constructs were transferred to another culture well, and 400 µL dimethyl sulphoxide (DMSO) was added to dissolve the formazan crystals. After centrifugation of the resulting solution at 13,000 rpm for 2 min to remove the scaffold debris, 200 µL supernatant was pipetted and placed in a 96-well plate. The optical density of the solution was recorded using a Microplate Reader (Bio-Rad, model 680, USA) at a wavelength of 570 nm. Three parallel samples were carried out.

### Animal test

For *in vivo* animal tests, the constructs were covered with silicone membranes, which functioned as a temporary epidermis, to create a bilayer dermal equivalent. Three groups were prepared for *in vivo* animal study, i.e. GAS, Blank/BM-MSCs and GAS/BM-MSCs constructs. The animal experiments were approved by the committee on animal experimentation of Zhejiang University and carried out in accordance with the guidelines for animal experimentation. As the most commonly used laboratory animal, rats have eccrine sweat glands exclusively present in the pads of their paws, which was selected as the locus of transplantation in this study. Briefly, female SD rats weighing 200 g were anesthetized by intraperitoneal injection of 4% (w/w) pentobarbital solution (0.2 mg/100 g body) in 0.01 M PBS. Both the hind paws of the rats were sterilized with 5% povidone-iodine (PVP-I). Using a scale, the area for incision was precisely measured and marked on both paws. The wounds with a diameter of 5 mm were created using forcep and scissor. After the incisional wounds were created on the paws, 3 groups of samples were implanted. Then the edges of scaffolds were carefully sutured. After that, it was covered with a dressing bandage. Special neck collars were designed to fix the rats and prevent from destroying the implanted samples. Totally 30 SD rats were used in the surgery. At the scheduled time points post-surgery, the macroscopic appearance of the wounds was recorded with a digital camera (Nikon D5200, Japan). The tissue samples at each time interval were harvested, and fixed in 4% (W/W) formaldehyde solution for paraffin section or frozen in liquid nitrogen for real-time quantitative PCR (RT-qPCR) and western-blotting (WB) analyses. At least three parallel rats were used for each group of animal test.

### Histological and immunohistological examination

For histological analyses, the harvested specimens were fixed in 4% paraformaldehyde at 4 °C overnight, and then dehydrated with a graded series of ethanol and embedded in paraffin. The sectioned samples with a thickness of 5 µm were then stained with hematoxylin-eosin (HE) and visualized using an optical microscope (IX81, Olympus, Japan).

To investigate the differentiation behaviors of BM-MSCs, the key differentiation markers of sweat glands, i.e. cytokeratin 8 (CK8) and cytokeratin 14 (CK14) were detected by immunohistochemistry. The paraffin sections (5 µm) were deparaffinized and washed three times in PBS (pH7.4) for 5 min, and then blocked with 5% serum for 30 min. The slides were subsequently exposed to rat anti-CK8 primary antibody (1:50, Abcam, Cambridge, UK) and rat anti-CK14 primary antibody (1:50, Abcam, Cambridge, UK) at 4 °C overnight, respectively. After rinsed three times with PBS, the slides were incubated with goat anti-rat secondary antibodies (1:200, Dako, CA, USA) at 37 °C for 20–30 min, and further developed with 3, 30-diaminobenzidine tetrahydrochloride (DAB) solution and finally counterstained with hematoxylin. Positive staining was indicated by a brown color observed under an optical microscope (IX81, Olympus, Japan).

### RT-qPCR analysis

Both the *in vitro* and *in vivo* samples were analyzed using RT-qPCR. For the *in vitro* measurement, different groups of scaffold cultured for specific time periods were harvested. Similarly for the *in vitro* measurement, the *in vivo* samples were harvested after euthanizing the rats at each predetermined time periods. Total RNAs of the samples were extracted by using Trizol (Invitrogen, USA) according to the manufacturer’s instructions and quantified by a biophotometer (Eppendorf, Germany). One μg of total RNA was reverse transcribed to produce complementary DNA (cDNA) with an omniscript RT kit (Qiagen). The resultant cDNA was used as a template for subsequent PCR amplification. The primer sequences used in this study are listed in Supplementary Table S1. The 18S ribosomal subunit (18S) was used as an endogenous reference housekeeping gene. The RT-qPCR was performed using the Power SYBR Master Mix (Invitrogen, USA) kit and CFX384 qPCR system (Bio-Rad, USA). The relative gene expression values were calculated using a comparative DDCT (threshold cycle) method and normalized to the housekeeping gene. Three parallel samples were carried out for each marker and each time point.

### Western blotting analysis

Western blotting (WB) analysis was further performed to quantify the expression of the sweat gland markers at the protein level. For WB analysis, both the *in vitro and in vivo* samples were harvested and frozen in liquid nitrogen and stored until use. The frozen samples were completely homogenized in radio immunoprecipitation assay (RIPA) lysis buffer (150 mM sodium chloride, 0.5% Triton X-100, 0.5% sodium deoxycholate, 0.1% sodium dodecyl sulfate (SDS), 50 mM Tris, pH 8.0) with protease inhibitors. The lysates were then clarified by centrifuging at 12,000 rpm for 15 min at 4 °C and separated on SDS-PAGE. After being transferred to a poly(vinylidene fluoride) (PVDF) membrane (Millipore, MA, USA), the proteins were incubated overnight with antibodies and detected using an enhanced chemiluminescence (ECL Western Blotting Substrate, Pierce, USA) system. The specific antibodies used for this experiment were Rat anti-CEA (Abcam, 1:200), CK14 (Abcam, 1:1000) and CK8 (1:800) primary antibodies, respectively.

### Statistical analysis

Data are expressed as mean ± standard deviation (SD). Statistical analysis was performed by two-tailed student’s t-tests between two groups or by one-way ANOVA between more groups. The significant level was set at p < 0.05.

### Data availability

All data generated or analysed during this study are included in this published article and its Supplementary Information files.

## Electronic supplementary material

Supplementary Information
