# Supplementary material for: Gene-activated matrix/bone marrow-derived mesenchymal stem cells constructs regenerate sweat glands-like structure in vivo
Source: Sci Rep. 2017 Dec 15;7:17630. doi: 10.1038/s41598-017-17967-x (PMC5732266; doi:10.1038/s41598-017-17967-x)
Supplement: Supplementary file 1 — Supplementary Information [file 41598_2017_17967_MOESM1_ESM.doc]

**Supplementary information**

**Gene-activated matrix/bone marrow-derived mesenchymal stem cells constructs regenerate sweat glands-like structure *in vivo***

Pranish Kolakshyapati^1 #^, Xiuyuan Li^1^ ^#^, Chunye Chen^2 #^, Mingxia Zhang^2^, Weiqiang Tang ^2 *^, Lie Ma^1 *^, Changyou Gao^1^

1 MOE Key Laboratory of Macromolecular Synthesis and Functionalization, Department of Polymer Science and Engineering, Zhejiang University, Hangzhou 310027, P.R. China

2 Department of Plastic Surgery, The Fourth Affiliated Hospital, College of Medicine, Zhejiang University, Yiwu, 322000, P.R. China

# These authors contributed equally to this work

* Corresponding authors: [liema@zju.edu.cn](mailto:liema@zju.edu.cn); [tanweixxxx@163.com](mailto:tanweixxxx@163.com)

1. **Characterizations of BM-MSCs**


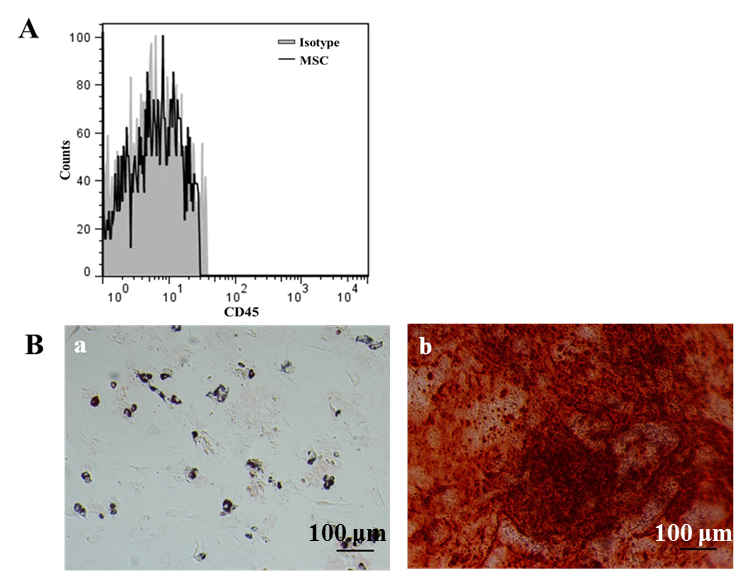


**Fig. S1** (A) Flow cytometry analysis of the isolated MSCs. (B) in vitro multidifferentiation potential. MSCs were stained with Oil Red O solution (a) and Alizarin Red S solution (b), respectively. Scale bar indicates 100 μm.

1. **The viabilities of BM-MSCs in different scaffolds**

**Fig. S2** Viability of BM-MSCs being cultured *in vitro* in Blank/BM-MSCs and GAS/BM-MSCs constructs as a function of time (n=3)

1. **Densitometry analysis of western blotting results**

The western blot images were quantified using Image J software. Firstly, the blot images were imported into the software and the contrast was adjusted such that the bands were clearly visible on the blot image. Then the area around each band was selected and the background intensity was subtracted from the blot image. Bands were then selected by drawing a tight boundary around them and the grayscale value of the selected bands was then measured and exported for carrying out further statistical analyses.

**
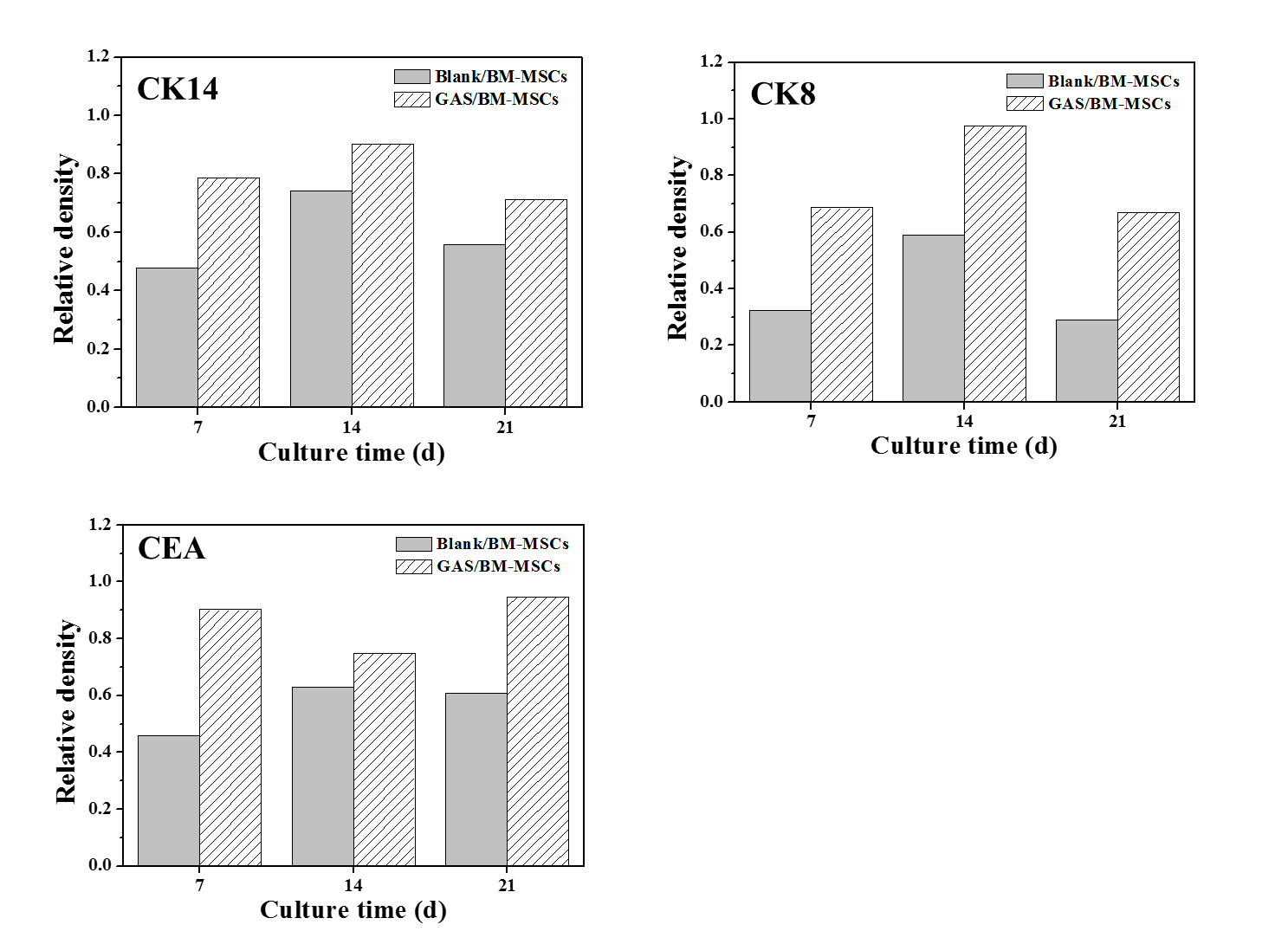
**

**Fig. S3** The densitometry analysis of the western blotting results in Figure 4.

1. **Immunohistochemical stainings of the in vitro cultured constructs**


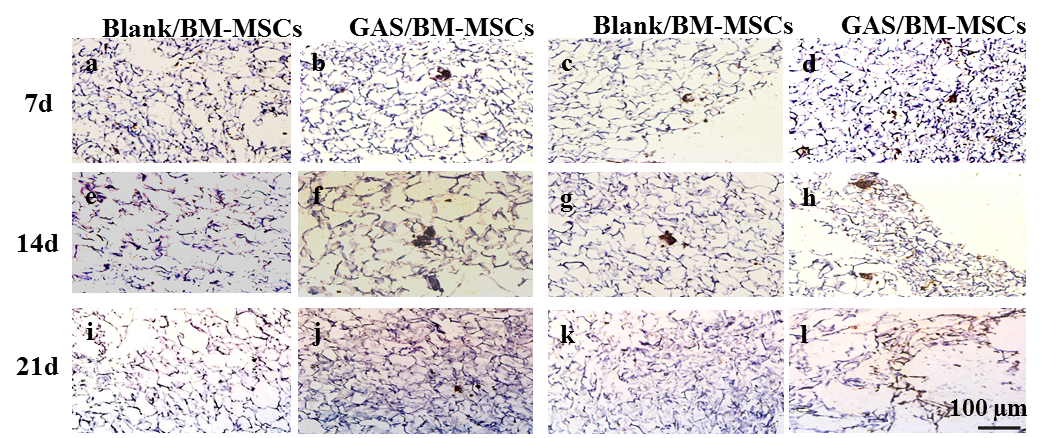


**Fig. S4** Immunohistochemical staining of CK8 (a, b, e, f, i, j) and CK14 (c, d, g, h, k, l) on Blank/BM-MSCs and GAM/BM-MSCs constructs after being cultured for 7, 14 and 21 days.

1. **Macroscopic appearance of rat paw**


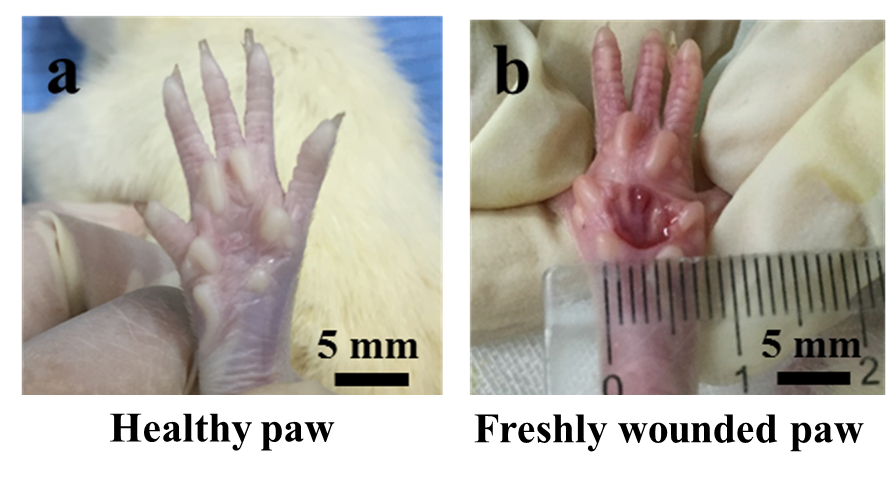


**Fig. S5** Macroscopic appearance of the healthy paw (a) and the freshly wounded paw (b).

1. **Overview pictures of H&E stainings**

**
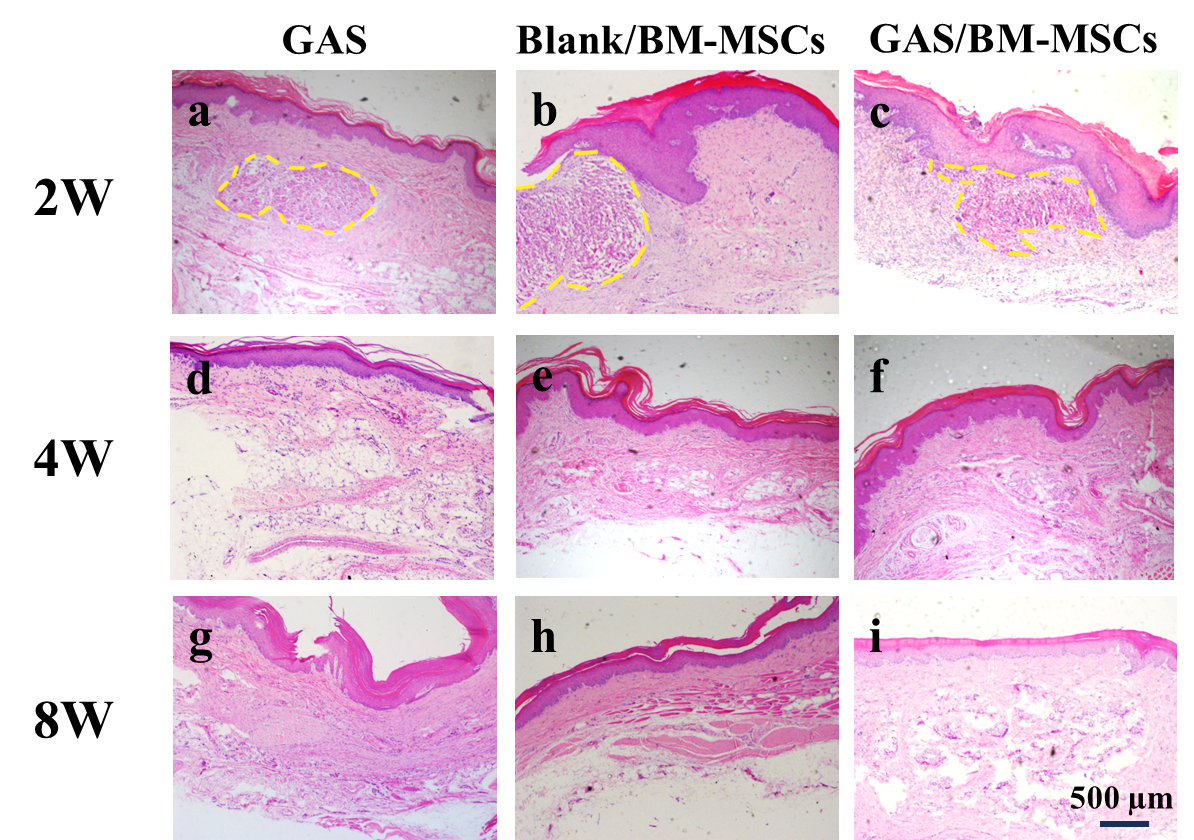
**

**Fig. S6** Overview pictures of the H&E stainings of the wounds treated by GAS (a, d, g), Blank/BM-MSCs (b, e, h) and GAS/BM-MSCs constructs (c, f, i) for 2w, 4w and 8w post-surgery, respectively. The regions labeled by yellow dash lines indicate the scaffold residues.


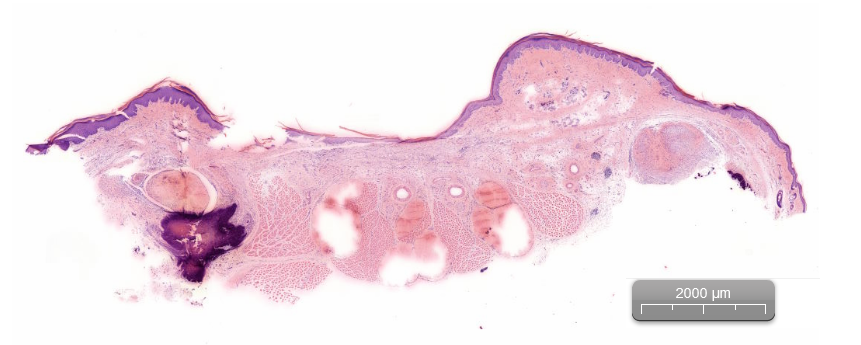


**Fig. S7** A complete scan of tissue slide taken from paw at 2 weeks after injuring. In this slide scan, the discontinuous epidermis as well as the wound can be observed clearly.

1. **H&E staining and immunohistochemical staining of native skin**


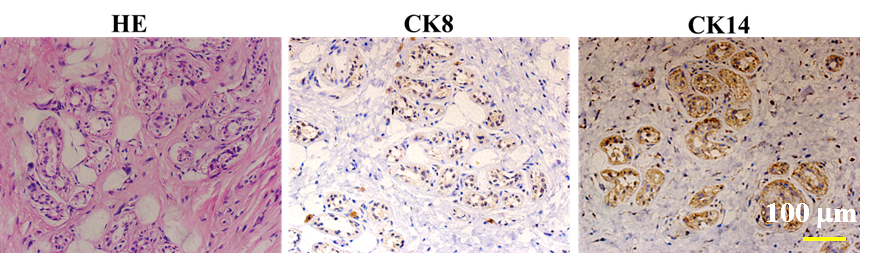


**Fig. S8** H&E staining and immunohistochemical staining of CK8 and CK14 of native skin.

1. **Densitometry analysis of western blotting results**


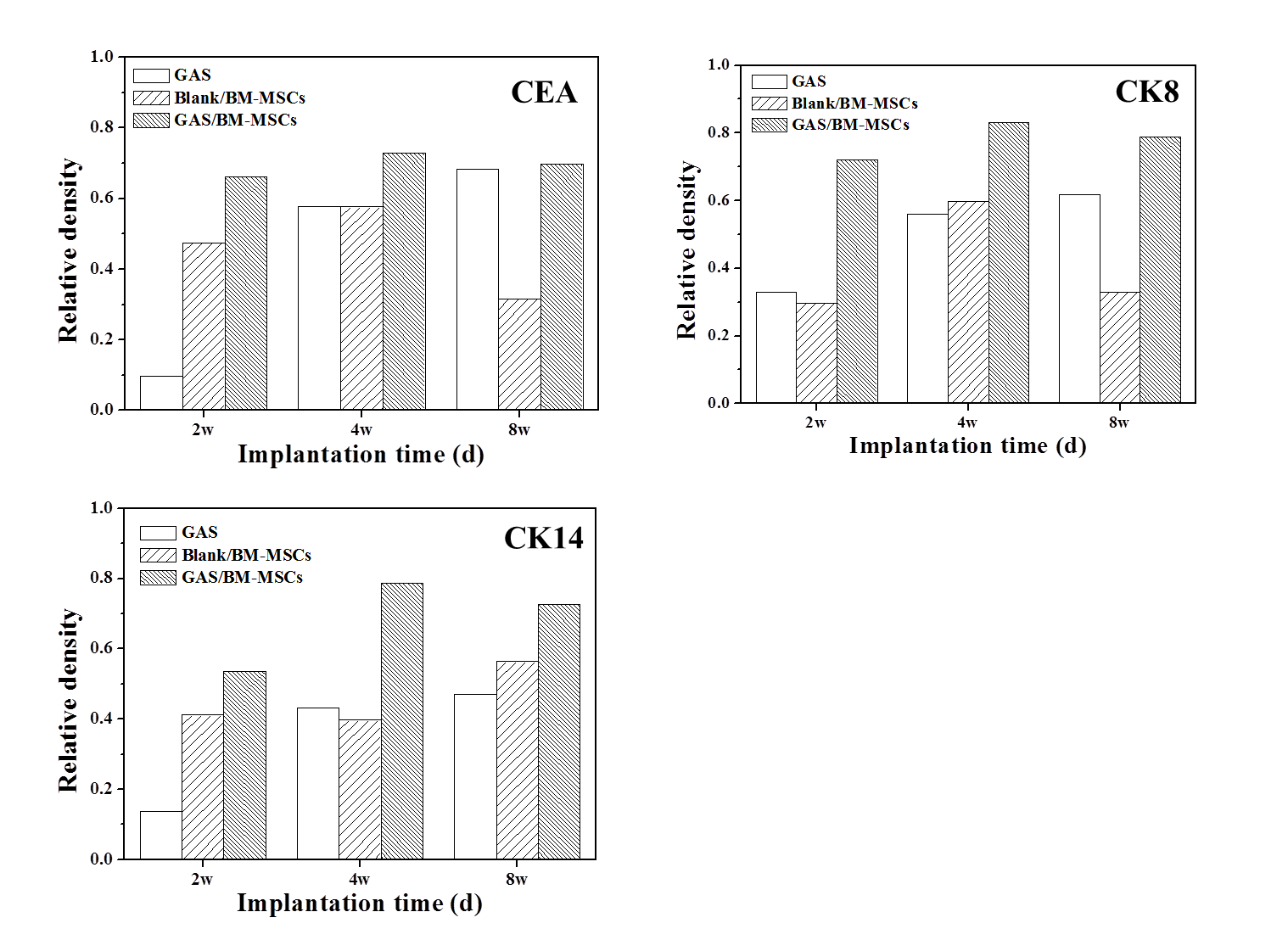


**Fig. S9** The densitometry analysis of the western blotting results in Figure 10.

1. **Transfection efficiency of the released DNA**

The transfection efficiency of the released DNA complexes was studied by using the pDNA-eGFP and MSCs. After the cells were seeded at a density of 2×10^4^ cells per scaffold and cultured for 24 h, the DNA complexes released at different time intervals were added with a concentration of 1 mg DNA per 10^4^ cells. The transfection efficiency was analyzed by a flow cytometry (FCM, BD Bioscience). The freshly fabricated DNA complexes were used as the control.

**Fig. S10** The transfection efficiency of the Lipofectamine 2000/pDNA-EGF complexes released from GAS as a function of the releasing time.

| **Table S1** Primers used for RT-qPCR | | | | |
| --- | --- | --- | --- | --- |
| Gene | Genebank Accession | Primer Sequences(5'to3') | Size (bp) | Annealing (ºC) |
| Rat Krt8 | NM_199370.1 | CCCTGAACAACAAGTTCGCCTCT | 83 | 64 |
|  |  | GCTCCATTTGGTCTCCAGCATCT |  |  |
| Rat Krt14 | NM_001008751.1 | CCGCAAGGATGCCGAAGA | 165 | 64 |
|  |  | GCTTTCATGCTGAGCTGGGACT |  |  |
| Rat CEA | NM_012525.1 | GGGAAGCGTTCTTCTCCTCGTTCA | 111 | 64 |
|  |  | GCTGTTCTGTATCGGGCTATCTCA |  |  |
| Rat 18s | M11188 | GAATTCCCAGTAAGTGCGGGTCATA CGAGGGCCTCACTAAACCATC | 105 | 64 |
